# Increased Expression of Phosphatidylcholine (16:0/18:1) and (16:0/18:2) in Thyroid Papillary Cancer

**DOI:** 10.1371/journal.pone.0048873

**Published:** 2012-11-06

**Authors:** Seiji Ishikawa, Ichiro Tateya, Takahiro Hayasaka, Noritaka Masaki, Yoshinori Takizawa, Satoshi Ohno, Tsuyoshi Kojima, Yoshiharu Kitani, Morimasa Kitamura, Shigeru Hirano, Mitsutoshi Setou, Juichi Ito

**Affiliations:** 1 Department of Otolaryngology-Head and Neck Surgery, Graduate School of Medicine, Kyoto University, Kyoto, Japan; 2 Department of Cell Biology and Anatomy, Hamamatsu University School of Medicine, Hamamatsu, Japan; 3 Department of Otolaryngology/Head and Neck Surgery, Hamamatsu University School of Medicine, Hamamatsu, Japan; Harvard Medical School, United States of America

## Abstract

A good prognosis can be expected for most, but not all, cases of thyroid papillary cancer. Numerous molecular studies have demonstrated beneficial treatment and prognostic factors in various molecular markers. Whereas most previous reports have focused on genomics and proteomics, few have focused on lipidomics. With the advent of mass spectrometry (MS), it has become possible to identify many types of molecules, and this analytical tool has become critical in the field of omics. Recently, imaging mass spectrometry (IMS) was developed. After a simple pretreatment process, IMS can be used to examine tissue sections on glass slides with location information.

Here, we conducted an IMS analysis of seven cases of thyroid papillary cancer by comparison of cancerous with normal tissues, focusing on the distribution of phospholipids. We identified that phosphatidylcholine (16:0/18:1) and (16:0/18:2) and sphingomyelin (d18:0/16:1) are significantly higher in thyroid papillary cancer than in normal thyroid tissue as determined by tandem mass (MS/MS) analysis. These distributional differences may be associated with the biological behavior of thyroid papillary cancer.

## Introduction

Thyroid cancer is the most common malignant tumor in the head and neck region. The histological types of thyroid cancer vary, and include papillary carcinoma (80% of all thyroid cancer cases), follicular carcinoma, medullary carcinoma, and undifferentiated carcinoma. Prognosis also varies depending on histological type. Undifferentiated carcinoma has a poor prognosis, with a 10-year survival rate of 10–20% or less, whereas patients with other histological types, such as papillary carcinoma, follicular carcinoma, and medullary carcinoma, can expect good outcomes with a 10-year survival rate of 90%, 90%, and 70–80%, respectively [1]. However, even cases of papillary carcinoma can fail to be controlled due to distant metastasis or anaplastic transformation. It will be necessary to reliably predict anaplastic transformation before it occurs, and to identify cases of poor prognosis among papillary carcinomas, in order to improve the overall prognosis of thyroid cancer.

Developments in genomics and molecular biology have shed light on pathogenic mechanisms related to thyroid cancer [2]. Great efforts have been made to identify genes and biomolecules that are differentially expressed in cancerous tissues, which can be utilized as biomarkers to elucidate thyroid cancer pathogenesis and guide appropriate and targeted molecular therapies [3], [4], [5]. Several candidate genes (for TSH receptors, RET/PTC, Ras, BRAF, p53) in the development of different types of thyroid cancer [2] have been identified thus far. In addition, some attempts have been made to utilize proteomics as a tool of discovery for thyroid neoplasms. Lewis and co-workers reported a difference in protein expression between papillary thyroid carcinoma and normal thyroid tissue using mass spectrometry (MS) [6]. However, the mechanism of malignant transformation is not well understood, especially at the protein level.

Lipids are associated with cell membrane structure, proliferation [7], differentiation, metabolic regulation, inflammation [8], and immunity. It is important to understand the relationship between tumor and lipids in diagnosis and treatment. Lipids, especially phospholipids (PLs), play important roles in the composition of the cell membrane. It is generally accepted that membrane characteristics are determined by the components of PL species, and the composition of these species is strictly determined by the components of fatty acid species [9], [10]. A few reports conducted to date have focused on lipids, especially binding fatty acids in head and neck cancer; however, to date, no method has been developed that enables the detection of binding fatty acids in PLs.

Imaging mass spectrometry (IMS) is a powerful, newly developed tool that identifies the distribution of known/unknown molecules on a tissue section [11], [12], [13]. Laser scanning enables precise, two-dimensional MS on glass slides. Currently, IMS is the only tool that allows for visualization of the binding of fatty acids to PLs on tissue sections, and this next-generation approach is attracting substantial attention.

The purpose of the present study was to use IMS to elucidate which PL-bound fatty acids were the main components of cell membranes, and in particular, which ones were expressed at relatively high levels in thyroid papillary cancer. This study was the first to investigate cases of PLs in thyroid cancer using IMS analysis, and the first to successfully identify PLs that are highly expressed in thyroid cancer.

## Results

### 1. IMS analysis of case 1

**Figure 1:**
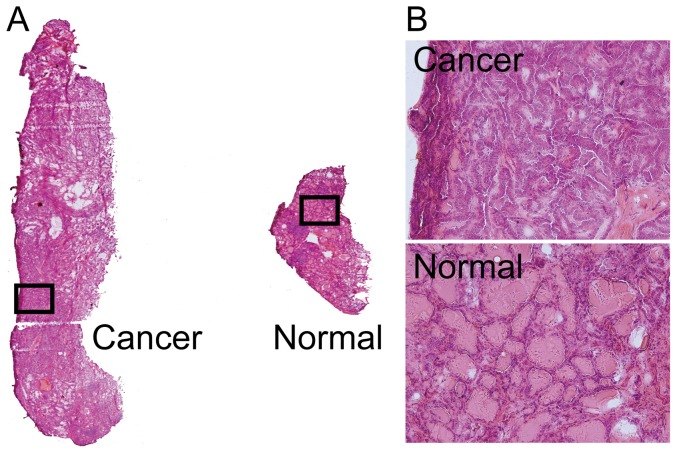
HE-stained section of case 1. (A) Thyroid papillary cancer tissue was localized on the left, and normal thyroid tissue was localized on the right (original magnification 40×). The stromal region was excluded. The ROI was determined from the corresponding HE-staining results. The black boxes indicate the representative region of cancer and normal thyroid tissue. (B) Magnified representative regions of cancer and normal tissue (original magnification 200×). The cancer cells had a high cytoplasmic ratio and displayed nuclear features characteristic of papillary thyroid cancer. Histologic findings of thyroid papillary cancer consisted of columnar thyroidal epithelium set in papillary projection. The normal thyroid tissue is composed of many spherical hollow sacs called thyroid follicles.

The regions of interest (ROI) in cancer and normal regions were defined according to hematoxylin and eosin (HE)-staining results of a tissue section adjacent to the section used for IMS analysis. Figure 1A provides HE-staining results for case 1 while Figure 1B shows magnified representative regions of cancer and normal tissue. The cancer cells had a high cytoplasmic ratio and displayed nuclear features characteristic of papillary thyroid cancer. Histologic findings of thyroid papillary cancer consisted of columnar thyroidal epithelium set in papillary projection. The normal thyroid tissue is composed of many spherical hollow sacs called thyroid follicles.

**Figure 2:**
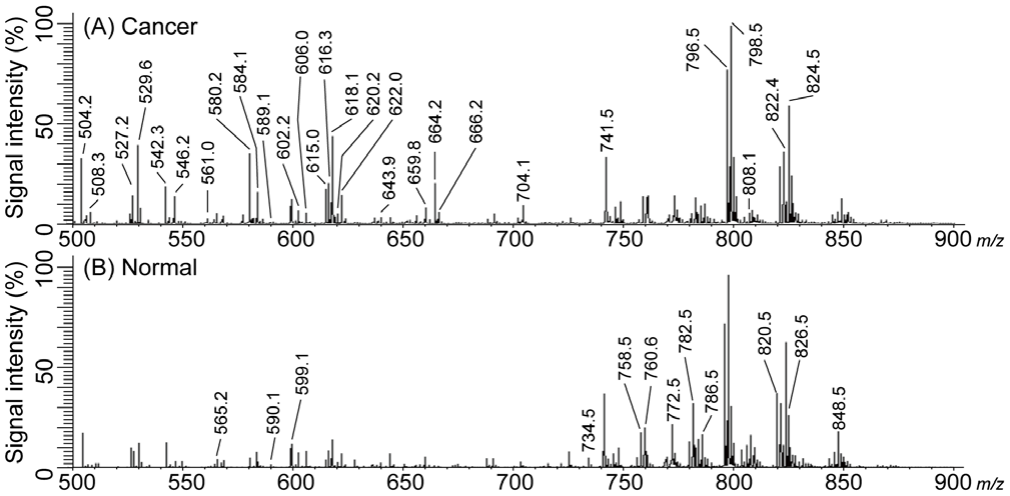
Averaged spectrum for case 1. (A) Spectrum of the cancer region and (B) spectrum of normal region. Each spectrum was averaged from the ROI of cancer and normal tissue in Figure 1. Each number shown in Table 1 was assigned using these spectra.

Figure 2 shows spectra obtained from case 1 tissue with panels A and B derived from cancer and normal regions, respectively. Both spectrums are average spectrums, and were obtained from ROI in cancer and normal tissue. The number of calculated points in the cancer and normal region was 1425 and 258, respectively. The horizontal axis indicates the mass-to-charge ratio (*m/z*) and the vertical axis indicates the relative abundance of the ion. The most intense ion is assigned an abundance of 100, and is referred to as the base peak. Most ions formed in a mass spectrometry have a single charge, so that the *m/z* value is equivalent to the mass itself.

Table 1 shows the top 50 peak picking results for case 1 (excluding isotopic peaks) that were statistically analyzed. Cancer and normal intensity means the average (± standard error) intensity that was divided by the scanning point in cancer and normal regions. Welch t-test was performed between the mean intensity of cancer and normal regions. The *m/z* values in Table 1 are listed in order of their intensity in the cancer region. The number of the *m/z* values without isotopic peaks was 40, and the number of the values with significant differences was 26. All *m/z* values in Table 1 were assigned using the spectrum shown in Figure 2A and B.

**Table 1:** Peak picking and statistical analysis of mass spectra in case 1.

*m/z* value	Cancer	Normal	P value
798.5	37.1±1.2	28.5±2.6	3.3E-03
824.5	26.7±0.8	26.6±2.4	9.7E-01
796.5	25.1±0.9	17.7±1.7	1.0E-04
618.1	20.4±1.0	5.1±0.6	4.3E-33
529.6	17.0±1.0	3.7±0.4	2.3E-29
580.2	16.2±1.1	2.0±0.3	7.1E-34
822.4	15.9±0.5	13.4±1.3	6.7E-02
504.2	15.6±1.1	6.7±0.8	5.7E-11
741.5	14.0±0.5	11.1±1.0	8.5E-03
664.2	9.5±0.9	0.3±0.1	7.9E-23
616.3	9.0±1.0	4.1±1.7	1.6E-02
826.5	8.7±0.3	8.9±0.9	8.4E-01
820.5	7.8±0.3	9.0±1.1	2.6E-01
584.1	7.4±0.7	1.4±0.2	3.9E-17
622.0	6.7±0.6	1.6±0.3	9.6E-14
760.6	6.5±0.4	7.7±1.0	2.4E-01
758.5	6.3±0.4	7.6±0.9	1.8E-01
527.2	6.2±0.6	3.8±0.5	3.1E-03
782.5	5.5±0.2	12.7±1.3	7.1E-08
542.3	5.5±0.5	0.2±0.0	6.5E-26
772.5	5.4±0.2	12.4±1.9	5.2E-04
615.0	5.4±0.2	1.3±0.1	1.8E-51
848.5	5.0±0.2	8.6±1.0	6.0E-04
546.2	4.8±0.4	0.4±0.1	2.2E-22
786.5	4.6±0.3	7.2±0.9	3.6E-03
704.1	4.0±0.3	0.5±0.1	1.8E-25
599.1	3.9±0.1	3.9±0.3	9.8E-01
659.8	3.7±0.3	1.3±0.2	3.2E-09
666.2	2.7±0.3	0.1±0.0	1.1E-18
508.3	2.6±0.3	0.2±0.0	3.3E-16
606.0	2.5±0.3	1.7±0.3	4.2E-02
808.1	2.5±0.2	2.1±0.7	6.0E-01
602.2	2.3±0.2	1.6±0.3	4.6E-02
565.2	2.0±0.2	2.1±0.3	8.8E-01
643.9	1.3±0.2	1.2±0.2	6.2E-01
734.5	0.7±0.1	3.2±0.7	1.8E-04
561.0	0.7±0.1	0.0±0.0	2.7E-15
620.2	0.3±0.1	0.1±0.0	2.2E-03
590.1	0.2±0.0	0.2±0.0	6.2E-01
589.1	0.1±0.0	0.0±0.0	2.8E-04

### 2. Visualization of molecular distribution in thyroid tissue of case 1

**Figure 3:**
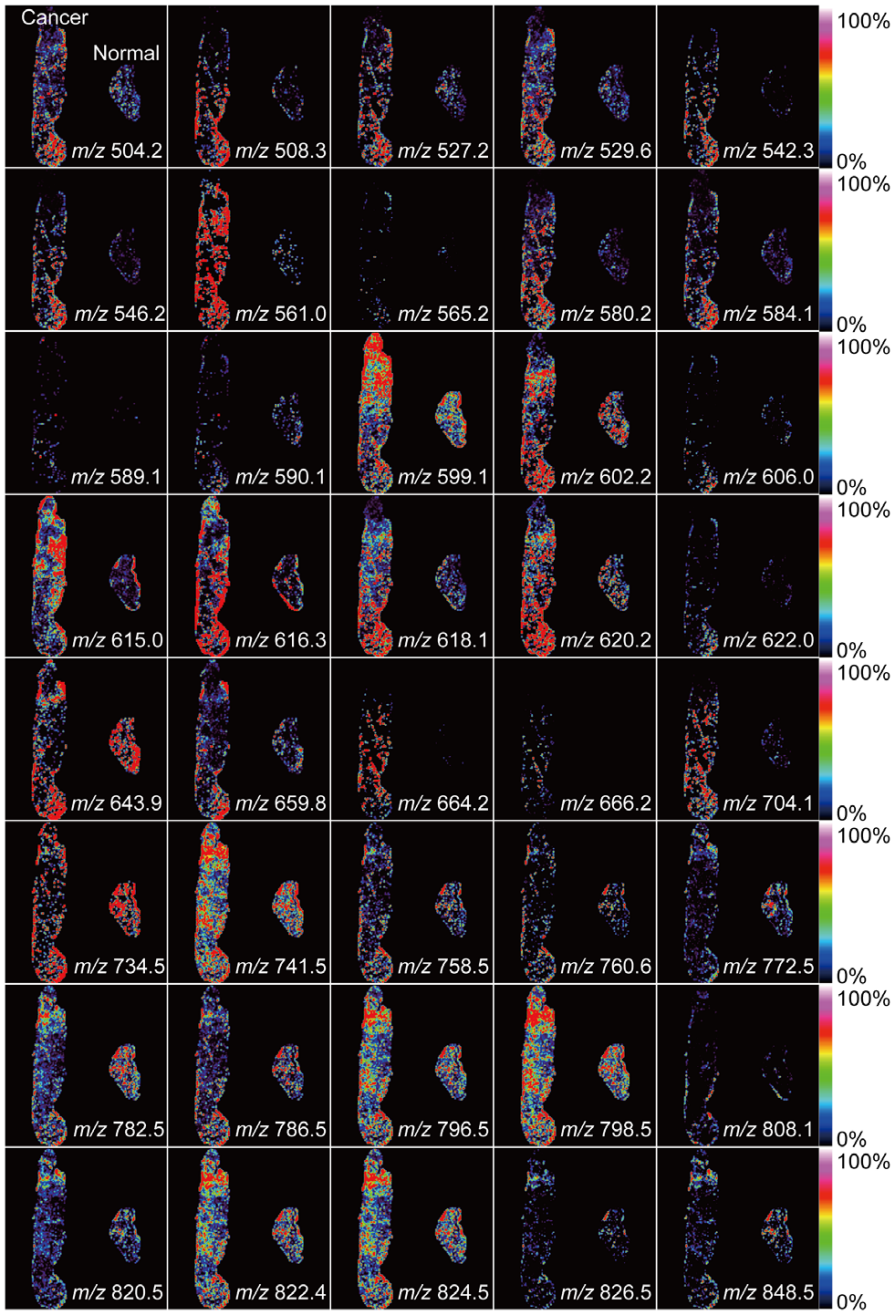
Visualization of molecular distribution in case 1. We visualized ion images corresponding to the results shown in Table 1. In all images, the cancer tissue is on the left and normal tissue is on the right. The distribution of the intensity for each *m/z* value was not constant in the cancer and normal thyroid tissue.

Figure 3 shows the ion image that was visualized using the *m/z* values shown in Table 1. The range of each ion color images was optimized manually, so the peaks have different color ranges. In general, malignant cellular proliferation was stimulated due to cell growth factors, which induce an increase in cell density and components of cancer cells such as PLs. Therefore, while the intensity of all *m/z* values should be higher in cancer regions, the intensity of some values (in particular, *m/z* 772.5, 782.5 and 848.5) in cancer regions was lower.

### 3. Comparison of results in all cases

In the same manner as case 1, peak picking and statistical analysis were performed on all cases. Table 2 shows the top 50 peak picking results and excludes isotopic peaks. The *m/z* values showing no significant differences were excluded. Three *m/z* values*, including *m/z* 798.5, 796.5, and 741.5, were found to be common for all cases.

**Table 2:** Overview of the *m/z* values that had higher expression levels in cancer regions of all cases.

Case 1	Case 2	Case 3	Case 4	Case 5	Case 6	Case 7
798.5*	798.5*	701.8	798.5*	798.5*	798.7*	798.4*
796.5*	741.5*	676.8	796.5*	741.5*	796.7*	824.4
618.1	796.5*	675.9	820.5	772.5	824.7	796.4*
529.6	824.5	700.8	822.5	796.5*	822.6	822.4
580.2	772.5	704.8	772.5	824.5	826.7	820.4
504.2	760.6	798.5*	741.5*	599.0	820.6	760.5
741.5*	826.6	796.4*	826.5	820.5	748.3	826.5
664.2	822.5	820.4	782.5	782.5	760.8	782.4
584.1	782.5	824.5		822.5	618.2	848.4
622.0	820.5	822.5		583.1	642.2	772.5
527.2	748.2	848.5		826.5	772.6	786.5
542.3	758.5	509.3		848.5	848.7	741.5*
615.0	848.5	741.6*		725.6	782.7	758.4
546.2	786.6	772.4		850.5	741.8*	846.4
704.1	725.6	825.5		756.5	580.3	784.5
659.8	784.6	846.4		748.2	758.7	850.5
666.2	691.3	850.5		844.4	774.3	748.1
508.3	851.6	849.5		846.4	786.8	810.5
561.0	846.4	844.4		760.6	784.7	844.3
620.2	810.5	782.5		780.5	746.4	788.5
589.1	849.5	893.6		808.5	644.1	734.5
	770.5	851.6		810.6	810.7	806.4
	734.6			828.5	504.3	703.5
	850.6			786.6	844.5	770.1
	844.4			758.6	828.6	534.5
	788.6			746.1	806.7	691.1
	534.6				528.3	725.5
	703.6				742.8	
	769.6				691.5	
	853.6				773.6	
	675.4				542.3	
					527.5	
					566.1	
					852.6	
					722.3	
					568.2	
					725.8	

**Figure 4:**
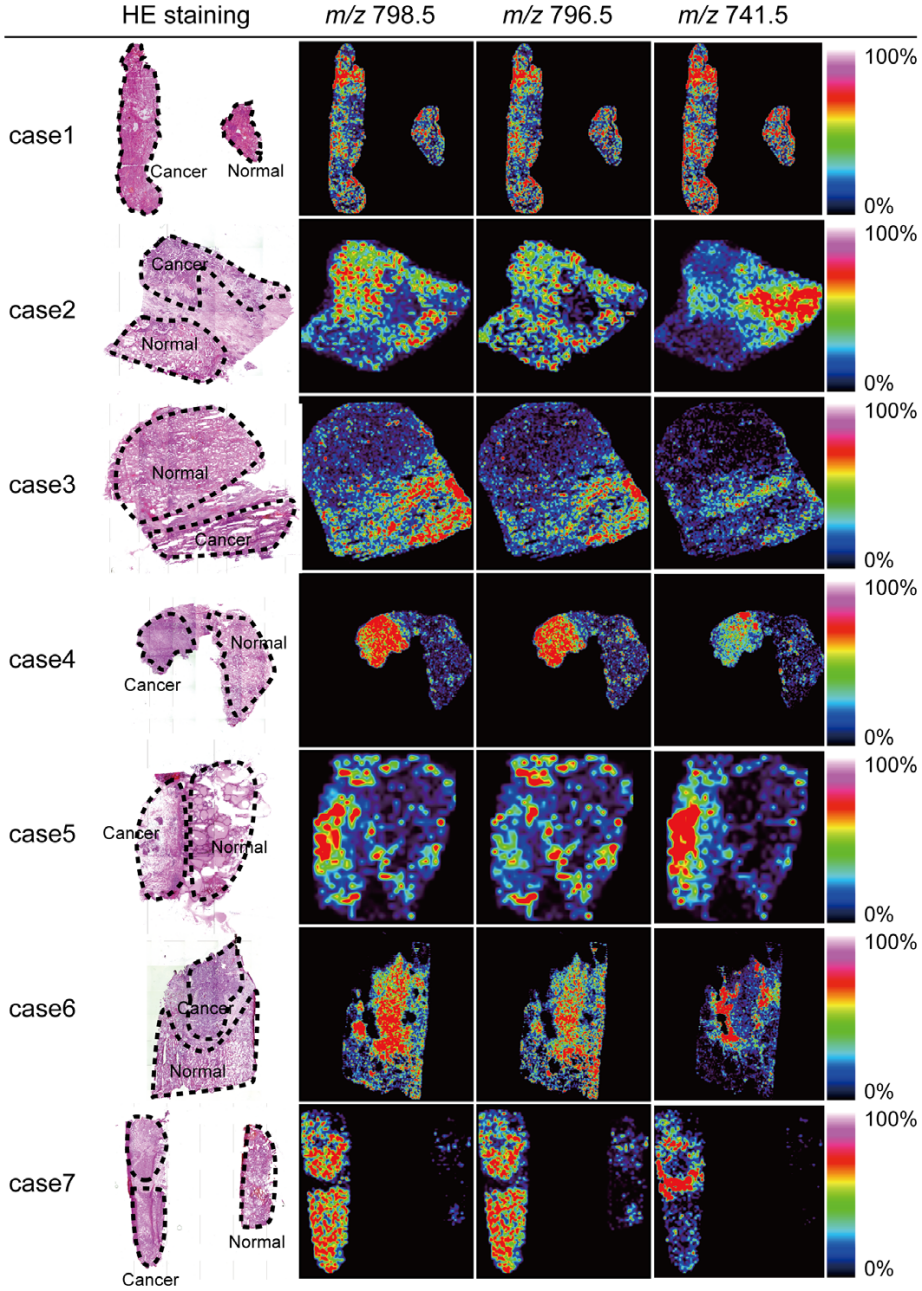
Visualization of molecular distribution of *m/z* values that were expressed to higher levels in cancer tissue from all cases. The ROI of each case is defined by a dashed line in HE-staining images. The intensity of all values in the cancer region was higher than in normal regions. The distribution of intensity in *m/z* 741.5 was different from the distribution of intensity in the other *m/z* values.

The common *m/z* values in all cases are displayed in Figure 4. ROIs of cancer and normal tissue in all cases were described in HE-stained results for all cases. The intensity of nearly all *m/z* values was higher in the cancer region compared to the normal thyroid region. Only the intensity distribution of *m/z* 741.5 differentiated them from the others.

### 4. Molecular identification

**Figure 5:**
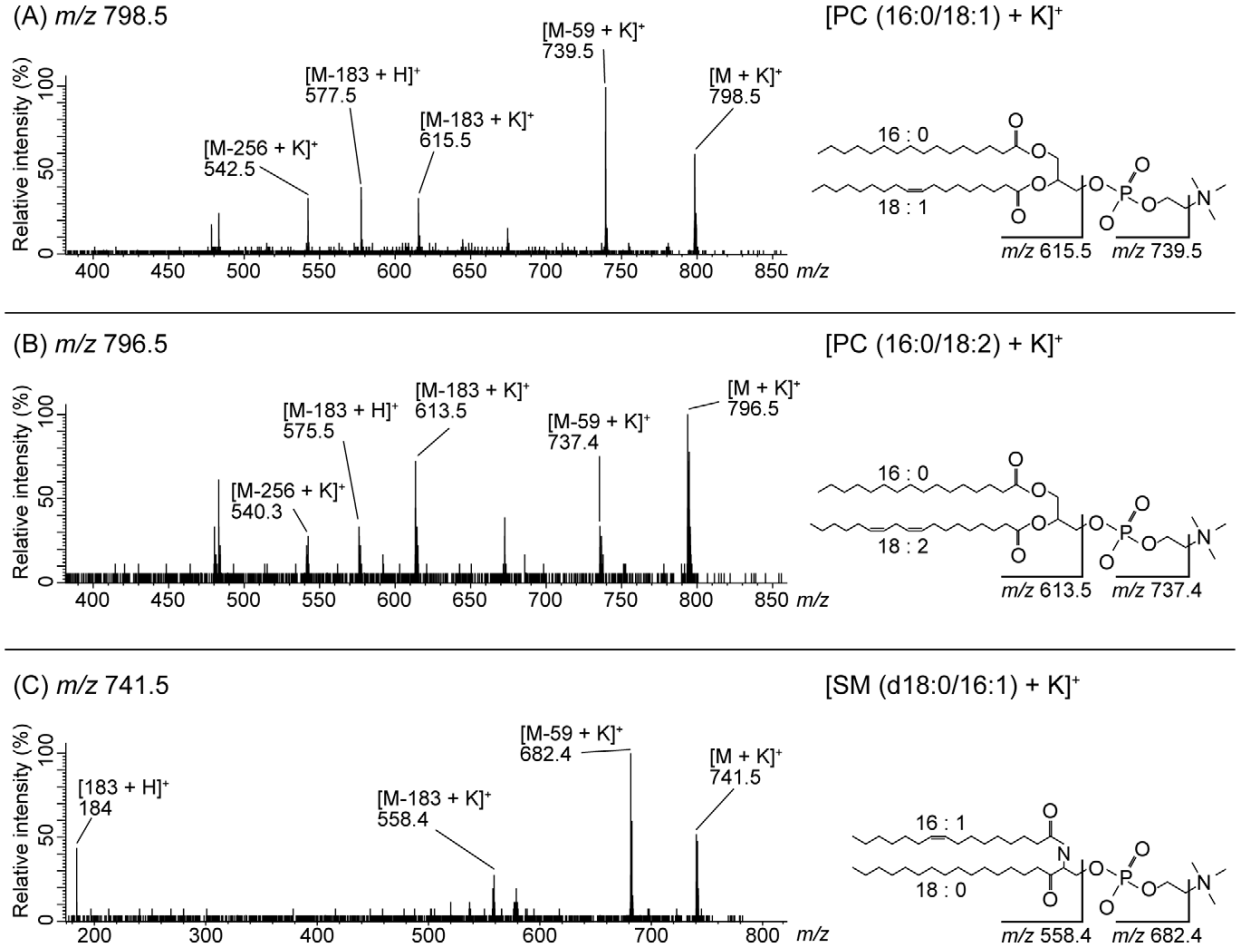
Identified molecules. (A) MS/MS data of *m/z* 798.5. The structure of one peak was analyzed. The product ion spectrum of *m/z* 798.5 as a precursor ion was obtained by MS/MS of thyroid papillary cancer region. This biomolecule was identified by neutral loss as [PC (16:0/18:1)+K]^+^. In the same manner, in (B) *m/z* 796.5 was identified as [PC (16:0/18:2)+K]^+^. (C) *m/z* 741.5 was identified as [SM (d18:0/16:1)+K]^+^.

The three common *m/z* values in all cases were subjected to tandem mass (MS/MS) analysis to identify the structures of the biomolecules associated with the precursor ions. (Figure 5). The Metabolite MS Search (http://www.hmdb.ca/labm/jsp/mlims/MSDbParent.jsp) was used for referencing.

In MS/MS for PLs with cations, certain characteristic fragment peaks are often detected. The peak at *m/z* 798.5 (Figure 5A) was identified as phosphatidylcholine (PC) due to the neutral loss of 59 Da and 183 Da during MS/MS, which is indicative of PC [14], [15]. Meanwhile, the neutral loss of 256 Da corresponded to palmitic acid. The type of cation adducted to a biomolecule is usually either a sodium or potassium ion, when the sample is obtained from biological tissue. A difference of 38.0 was observed between the *m/z* 577.5 and 615.5, which is consistent with the replacement of a potassium ion (molecular weight, 39.10) with a proton (molecular weight, 1.01). According to a Metabolite MS search, the peak at *m/z* 798.5 indicates [PC (16:0/18:1)+K]^+^. In the same manner, we concluded that *m/z* 796.5 corresponded to [PC (16:0/18:2)+K]^+^ (Figure 5B).

The results of *m/z* 741.5 showed peaks of *m/z* 682.4 and 558.4 (Figure 5C). The peak at *m/z* 682.4 corresponded to neutral loss of trimethylamine (59 Da), and the peak at *m/z* 577.5 corresponded to neutral loss of trimethylamine (59 Da) and cyclophosphate (124 Da). The peak at *m/z* 184 corresponded to trimethylamine (59 Da), cyclophosphate (124 Da) and a proton ion (1 Da). These results indicated that *m/z* 741.5 contained an alkali metal adduct phosphocholine; therefore, *m/z* 741.5 was a PC or sphingomyelin (SM) species. Applying the nitrogen rule to phospholipids, the odd nominal mass indicated SM due to the presence of an additional nitrogen in the sphingosine of SM. We thus concluded that *m/z* 741.5 was a SM species. The Metabolite MS search indeed suggested that *m/z* 741.5 corresponded to [SM (d18:0/16:1)+K]^+^.

## Discussion

For more than a century, pathological examinations have been the primary and most important tool for the diagnosis of cancerous regions. Cancer classification itself has been established based on the findings of classical staining methods such as HE staining, and such methods will continue to play a leading role in cancer diagnosis. However, the limitations of classification based on classical staining findings should be noted. It is often the case that patients with the same pathological diagnosis do not always have the same prognosis. Diagnoses are often made based on morphology. Using conventional pathological techniques, we can only conduct morphological observations, and it is difficult to reveal the details of components in tissue sections. *In situ* hybridization and/or immunohistochemistry analyses enable the analysis of the distribution of known molecules; however, it has remained impossible to examine the distribution of unknown molecules. For a detailed and accurate diagnosis, it is necessary to obtain information regarding components such as specific proteins and lipids in a sample.

As an important technique in the proteome generation post-genome era, MS has become widely used in numerous medical fields for the diagnosis and treatment of various diseases, including cancer. Numerous novel biomarkers have been identified thus far using MS, which has since been expanded to include IMS [11], [16], a technique that enables analysis and visualization of the distribution of individual biomolecules in any area of a tissue section [17]. Thus, this approach is potentially of great significance.

One IMS sequence creates a number of spectrums. To obtain valid information from these spectrums, we performed the addition of an adduct ion. In biological tissue, lipids tend to be positively charged by a proton, sodium, and a potassium ion, which indicates that the distribution of the positive ion influences the distribution of lipids; different values tend to be obtained in the IMS analysis results. In this study, we added potassium salt to the matrix based on reports of Sugiura and co-workers who selectively analyzed PC with different fatty acid compositions by the addition of potassium salt to the matrix solution [18]. When added to the matrix, the potassium salt solution caused a merging of various ion adducts (adducts with proton, sodium, and potassium) into one single potassiated species. This approach enabled us to reduce a number of peaks and made it easier to identify molecules of interest.

In the previous report, we demonstrated the feasibility of IMS as a tool for the analysis of pathological specimens [19]. We showed that IMS can be used to profile biological molecules, including subtypes of PLs. We focused on the distribution of PLs in thyroid papillary cancer. PLs are present as a constituent of the cell membrane and are also expressed in cancer tissue. The intensities of most *m/z* values in cancerous regions are higher than those in normal regions; however, the intensity of some values (*m/z* 772.5, 782.5 and 848.5) in cancer regions was lower than that in normal regions of tissue (see Figure 2 and Table 1). In general, malignant cellular proliferation was stimulated due to cell growth factors, which induce an increase in cell density and components of cancer cells such as PLs. PLs are comprised of numerous combinations of lipids that are based on their length, degree of acyl chain satiation, and the polar head group. Figure 2 suggests that these differences in intensity may arise from the distribution of the thyroid cancer-specific fatty acids that are attached to PLs.

In breast cancer, PLs, especially PCs, in cancer tissues were reported to have a relatively high level of linoleic acid (18:2) and low levels of stearic acid (18:0) [20] and oleic acid (18:1) [21], when compared with normal breast tissue. Luisa *et al.* identified the pattern in the PL pattern and class differences in breast cancer cells [22]. In their report, cancer cells showed a high relative abundance of PC (16:0/18:1) and PC (18:1/18:1) corresponding to [MH]^+^ at *m/z* 760 and 786. For SM, SM (18:1/16:0) corresponding to [MH]^+^ at *m/z* 703 was detected mainly in cancer cells.

Another previous report showed that the mRNA of 1-acylglycerol-3-phosphate-O-acyltransferase (AGPAT) 11 that efficiently uses LPA (18:1) as an acyl acceptor and fatty acid 18:1 as an acyl donor is significantly up-regulated in human breast and cervical cancer [23]. Our results show that the *m/z* 798.5 peaks contain fatty acid C18:1, and they are expressed strongly in thyroid cancer regions. While a previous study revealed the selection of specific fatty acid-binding PLs, we provide more information on the relative changes of PLs in thyroid papillary cancer.

In Figure 4, distribution of the intensity in *m/z* 741.5 corresponded to [SM (d18:0/16:1)+K]^+^ and is different from the intensity distribution in the others except for case 1 and 5. HE-staining results showed that the area in which *m/z* 741.5 intensity is expressed strongly mainly consisted of stromal and cancer regions. We reported that the intensity of expressed SM was higher in cancer and stromal regions than in normal regions in a study of colon cancer liver metastasis [19]. Our result was consistent with this report.

Stearoyl-CoA desaturase 1 (Scd1) is the rate limiting enzyme in the cellular synthesis of monounsaturated fatty acids, including C18:1, from saturated fatty acids. Falvella *et al.* reported that Scd1 gene overexpression is associated with hepatocarcinogenesis in mice [24]. Scaglia *et al.* reported that inhibition of Scd1 expression in human lung cancer cells impairs tumorigenesis, whereas the rate of apoptosis was elevated [25]. These reports indicate that Scd1 causes an increase in C18:1 in cancer tissue.

The type of fatty acid influences cell shape and cell membrane fluidity [9]. The alterations of cancer cell membrane fluidity may influence the biological behavior of cancer such as invasion/metastasis. IMS is the only tool that allows for the visualization of the binding of fatty acids to PLs on tissue sections. Recently, an increasing number of reports has focused on the relationship between pathological insults and PLs, including PCs [26], i.e., the remodeling pathway of PLs [27]. It is expected that IMS analysis will help to achieve a better understanding of the relationship between fatty acids and cancer mechanisms.

Using IMS, we directly profiled PC and SM expression from tissue samples. This explorative profiling of PL on the basis of IMS analysis provided results that emphasize the potential of IMS for pathological diagnoses. The potential application of IMS analysis in the clinical workflow has been suggested in a previous report [28]. Compared to conventional methods such as MS and immunohistochemistry, IMS has certain advantages as a clinical application. Sample purification and extraction is necessary prior to MS analysis. In addition, the performance of an antibody-based assay in immunohistochemistry is always degraded to easily observed changes in intensity or localization. On the other hand, IMS analysis requires only a simple pretreatment, i.e., matrix deposition and fixing the IMS condition settings. This means that time is not lost between sample collection and analysis. It is predicted that IMS will be readily introduced into the pathological examination setting.

Recent studies have provided evidence of the clinical benefits of IMS analysis, namely, that its profiles discriminate between other diseases and prostate cancer [29] and the HER2 status of breast cancer [30]. In these reports, IMS enabled the classification of morphological and diagnostic features. A recently developed variant of IMS analysis, referred to as “targeted imaging mass spectrometry” (TIMS), was described by Thiery and colleagues [31]. Such targeted analysis enabled the visualization of molecules of interest directly from the tissue section by the use of laser reactive photocleavable molecular tags attached to antibodies. This approach provides quantification by estimating the signal intensity, and an excellent signal-to-noise ratio in the resulting spectrums. It is important to note that this diagnosis was made on a single sample section; few cancer biopsies were available, and more would be needed to quantitate a biomarker by IHC. In the future, IMS analysis will provide new biomarkers and in turn, new pathological categories, and could therefore become a critical diagnostic tool in the clinical setting.

## Materials and Methods

### Ethics statement

Sample collection and archiving of patient data was performed using written informed consent, and was approved by the ethical committee of Kyoto University. This study was performed in accordance with the Kyoto University guidelines for pathological specimen handling.

### 1. Sample preparation

Seven Japanese patients who underwent routine thyroidectomy at Kyoto University Hospital were involved in the present study. Six women and one man were included and the average patient age was 52 years. There was no recurrence in all cases. Samples were obtained from a thyroid cancer section and adjacent normal tissue immediately after tumor resection. The pathological diagnosis was papillary thyroid carcinoma. The obtained tissue was frozen in liquid nitrogen immediately to minimize degradation and was kept at −80°C. The tissue sections were sliced to a thickness of 10 µm using a Cryostat (CM 1950; Leica, Wetzler, Germany). One section was mounted onto an indium-tin-oxide-coated (ITO) glass slide for IMS analysis. Another section adjacent to that used for IMS was mounted onto a glass slide (MAS coat; Matsunami, Osaka, Japan) for HE staining to identify cancer and normal thyroid regions.

### 2. Matrix deposition

The matrix solution was prepared by dissolving 50 mg 2, 5-dihydroxybenzoic acid (DHB; Bruker Daltonics, Leipzig, Germany) in 1 mL 70% methanol and 10 mM potassium acetate. DHB is a widely used matrix for low molecular weight molecules. The addition of potassium salt to the matrix solution caused a merging of various ion adducts into one single potassiated species. This approach enabled us to reduce the number of peaks and simplified the identification of molecules of interest. A thin matrix layer was applied to the surface of the tissue sections using a 0.2-mm nozzle airbrush (Procon Boy FWA Platinum; Mr. Hobby, Tokyo, Japan) maintained at 15 cm from the tissue surface. The total amount of matrix solution on each slide was 1 mL. The spraying technique enabled full matrix coverage over the entire tissue surface and facilitated the co-crystallization of the matrix and bio-molecules.

### 3. Imaging mass spectrometry analysis

The tissue sections were analyzed using a matrix-assisted laser desorption/ionization-time-of-flight/time-of-flight (MALDI-TOF/TOF)-type instrument, Ultraflex II TOF/TOF (Bruker Daltonics), which was equipped with a 355-nm Nd: YAG laser at 200 Hz repetition. Data were acquired in the positive-ion mode using an external calibration method with ions from DHB, angiotensin II and bradykinin. Their decomposition products covered from *m/z* 100 to 1200. Calibration proteins were deposited on the surfaces of sample materials. Each raster scan was automatically performed in the regions of cancer and normal tissue. The interval between data points was 100-µm, and 100 laser beam shots were irradiated on each data point. The mass spectrometry parameters were optimized manually to obtain the highest sensitivity with *m/z* values in the range of 400–900. All spectra were acquired automatically using flexImaging 2.1 software (Bruker Daltonics), and the file format was converted to enable analysis with Biomap (http://www.maldi-msi.org) and SIMtools software (in-house software; Shimadzu Corporation). The ion image was visualized using Biomap software.

### 4. Comparison of signal intensities between cancer and normal thyroid regions

IMS analysis results were integrated, and the ROI in cancer and normal thyroid tissues were defined corresponding to HE results using SIMtools software. The top 50 peaks excluding isotopic peaks were picked from ROI that was defined as cancer and normal thyroid region, and the statistical difference was determined by Welch t-test. Differences with p<0.01 were considered significant.

### 5. Comparison of results for all cases

We performed IMS and statistical analysis on all cases, and picked the *m/z* value that had significantly higher expression in cancer regions and was common in all cases.

### 6. Molecular identification

The common *m/z* values in all cases were employed for MS/MS analysis for molecule identification. MS/MS analysis was performed on tissue sections in the positive-ion mode using QSTAR Elite (Applied Biosystems/MDS Sciex, Foster City, CA, USA), a hybrid quadrupole/TOF mass spectrometer equipped with an orthogonal MALDI source and a pulsed Nd: YAG laser. Metabolite MS Search was used to determine the molecular species of PLs. The matrix solution was prepared in the same way as for IMS analysis. The mass spectrometry parameters were optimized manually to obtain the highest sensitivity with m/z values in the range of 100–850.
